# Differential roles of resident lung macrophages during control of murine alveolar and airway *Mycobacterium abscessus* infection

**DOI:** 10.3389/ftubr.2026.1781507

**Published:** 2026-06-11

**Authors:** Kelsey C. Haist, Jack H. Congel, Jodi M. Corley, Alma E. Ochoa, Jazalle McClendon, Patrick S. Hume, Jerry A. Nick, William J. Janssen, Kenneth C. Malcolm, Katherine B. Hisert

**Affiliations:** Department of Medicine, National Jewish Health, Denver, CO, United States

**Keywords:** bronchiectasis, chronic infection, macrophage, *Mycobacterium abscessus*, nontuberculous mycobacteria

## Abstract

**Introduction:**

*Mycobacterium abscessus* (*Mabsc*), a nontuberculous mycobacterium (NTM), is readily cleared from healthy lungs but can cause infections in immunocompromised individuals and individuals with chronic airways diseases that disrupt mucociliary clearance, such as cystic fibrosis and bronchiectasis. In bronchiectatic airways, *Mabsc* can persist despite robust immune cell recruitment, raising the possibility that, in addition to impaired mucociliary clearance, local pulmonary immune defects contribute to NTM susceptibility. Since chronic infections result from failed eradication of acute infection, we sought to determine whether immune cell responses critical for control of acute *Mabsc* infection in healthy lungs remain relevant when *Mabsc* infection occurs in obstructed airways, rather than in the alveoli, as occurs in bronchiectasis.

**Methods:**

Using an agar bead model of *Mabsc* infection that prolongs murine small airway infection, replicates factors associated with bronchiectasis, and mirrors pathology of human *Mabsc* lung disease, we tested the hypothesis that *Mabsc* infection in obstructed airways elicits a qualitatively different immune response that alveolar *Mabsc* infection. We compared myeloid cell responses in in the two infection models using flow cytometric analyses of lung cells, immunofluorescent imaging, and histopathologic evaluation of lung sections.

**Results:**

During the first 2 weeks of the bead model of *Mabsc* infection, absolute abundance of neutrophils in the lungs was significantly higher and relative abundance of recruited macrophages was lower than in the alveolar *Mabsc* infection model. Additionally, most resident alveolar macrophages (CD11c^+^ RAMs) in the bead model upregulated CD11b, a marker of inflammation, by 1-week post-infection and maintained high levels of CD11b expression at 3 weeks post infection despite infection occurring in airways, not alveoli. To understand the role of macrophage subsets in controlling *Mabsc* infection, clodronate liposomes were administered oropharyngeally to deplete RAMs. RAMs were essential for early control of alveolar *Mabsc* infection, but depletion of RAMs had no effect on control of *Mabsc* burden during bead infection.

**Conclusions:**

These studies demonstrate that murine *Mabsc* airway infection induced by obstructing small airways with *Mabsc* embedded in agar beads generates an immune milieu distinct from that induced by *Mabsc* infection in alveoli, altering the importance of different macrophage populations for control of infection.

## 1 Introduction

Nontuberculous mycobacteria (NTM) are opportunistic pathogens that are ubiquitous in the environment but are readily cleared by host mechanisms within the healthy human lung (1). However, people with chronic airways diseases, such as COPD, cystic fibrosis, and bronchiectasis, are at high risk of developing pulmonary infection with NTM species, especially *Mycobacterium avium* and *Mycobacterium abscessus (Mabsc)*, which account for the vast majority of NTM lung disease in these groups (2–6). Chronic infection with NTM causes progressive damage to the lungs and is a therapeutic challenge: treatment requires multiple antibiotics for months to years, is fraught with side effects, and often fails to clear the organism (6, 7). A better understanding of how healthy lungs protect against NTM lung infection, and whether local immune cell populations differ in patients with chronic airways diseases, could lead to testing of host-directed therapies to resolve NTM lung disease.

Despite being susceptible to opportunistic pathogens such as NTM, most people with chronic airways disease are not considered immunocompromised, as they do not generally develop systemic or extra-pulmonary infections. Chronic airway damage causes impaired mucociliary clearance, an independent risk factor for NTM lung disease (1, 8), which allows opportunistic bacteria to linger in the damaged airways, resulting in a robust immune cell infiltration composed primarily of neutrophils and recruited monocyte-derived macrophages (9–12). However, in many cases, these immune cells fail to clear the acute infection, and NTM lung disease becomes chronic. Thus, it thought that chronic airways diseases create local pulmonary host defense defects (13–16), which remain poorly defined. A major barrier to understanding the nature of these host defense defects is a lack of relevant animal models (17, 18). Recently, there has been increasing interest in use of bacteria embedded in agar beads to create murine models of prolonged infections with opportunistic bacteria (19–21). This method is appealing for the study of airways disease as intra-tracheal (or oropharyngeal) instillation of bacteria embedded in 100–200 µm diameter agar beads reliably lodge in small airways (19–21), with the anatomical distribution of beads mirroring the location of many infections in people with bronchiectasis (22–25). Other features of this model that reflect bronchiectasis include the finding that agar bead infections induce focal areas of hypoxia and expression of hypoxia-induced factor 1α (HIF-1α) in the lung (26), as well as decrease the rate of mucociliary transport (27). Importantly, the pathology observed at late time points in mice inoculated with *Mabsc* beads closely resembles pathology of *Mabsc* pulmonary disease in people with chronic airways diseases (28).

Although use of agar beads embedded with opportunistic bacteria has been used for several decades to generate extended pulmonary infections in immunocompetent mice (29–31), the mechanisms by which the bead model enhances duration of infection have not been well established. Specifically, very little is known regarding whether the nature of the immune niche of the small airways contributes to the prolonged nature of agar bead infections (28, 32). We sought to identify which immune responses are crucial for control of acute *Mabsc* alveolar infection in the healthy lung and then determine if these same immune responses are activated or present during *Mabsc* agar bead-based obstructive small airway infections. We compared myeloid cell responses (the relative and absolute abundance of resident alveolar macrophages (RAMs), recruited monocyte-derived macrophages, and neutrophils) and lung histopathology in mice infected oropharyngeally (o.p.) with *Mabsc* in suspension (alveolar infection) vs. *Mabsc* embedded in agar beads (obstructive small airway infection). Additionally, we selectively depleted RAMs to determine their role in initial control of *Mabsc* in the two models of infection. Our studies reveal that the murine model of *Mabsc* bead-based obstructive small airway infection, with features that resemble airway disease in people with bronchiectasis, is associated with a qualitatively different immune cell infiltration during the initial host response to infection as compared to alveolar *Mabsc* infection. Our findings suggest that macrophage heterogeneity contributes to variations in host-pathogen interactions, potentially contributing to whether *Mabsc* infections are cleared or become chronic.

## 2 Material and methods

### 2.1 Mouse experiments

All mice listed were approved by the Institutional Animal Care and Use Committee (IACUC) (Protocol number: AS2574-02-26) at National Jewish Health. WT C57BL/6J mice were obtained from the Jackson Laboratory and used at 8–12 weeks of age. Mice were inoculated via oropharyngeal aspiration with liposomes and/or *Mabsc* as described below. Oropharyngeal aspiration results in similar distribution of inocula in the lungs as intratracheal instillation and is considered by some to better reflect human clinical infection (33). Brieﬂy, mice were anesthetized with isoﬂurane and positioned vertically. The tongue was gently pulled out of the mouth laterally to cover the esophagus and minimize obstruction of the trachea. 50 µL of the inoculum was deposited in the oropharynx and then aspirated into the lungs during the mouse's subsequent inhalation. On indicated days post-infection, mice were euthanized via intraperitoneal injection of barbiturate solution (Fatal-Plus). For experiments with only planktonic bacteria, BAL was performed for flow cytometry, and whole lung was collected and homogenized to plate for colony forming units (CFUs). For experiments with only agar bead-embedded bacteria, the left lung main stem bronchus was tied off, and the left lung was removed for homogenization and plating for CFUs. Bronchoalveolar lavage (BAL) was performed on the right lung for ﬂow cytometry. For experiments with both planktonic and agar bead-embedded bacteria, the left lung main stem bronchus was tied off, and the left lung was removed for histology. BAL was performed on the right lung, which was then removed for homogenization and plating for CFUs or flow cytometry. We have compared CFUs between left and right sides of the lung post-infection and found them to be comparable.

### 2.2 *M. abscessus* planktonic infection (bacteria in suspension) to generate alveolar infection

*M. abscessus* (*Mabsc*) ATCC 19977 smooth variant was used for all experiments. Liquid aliquots of 7H9 Middlebrook media (Sigma-Aldrich) supplemented with 10% of the Middlebrook ADC (albumin/dextrose/catalase) growth supplement (Millipore Sigma) were inoculated with *Mabsc*, and cultures were incubated at 37 °C shaking at 225 rpm for 3 days. These cultures were then used to inoculate mice oropharyngeally with 1 × 10^6^ CFUs per mouse. Evaluation of BAL at days 1 and 3 post infection confirm that planktonic inoculum is delivered to the airspaces where it interacts with alveolar macrophages (Supplementary Figure S1).

### 2.3 Infection with *M. abscessus* embedded in agar beads that lodge in small airways

*M. abscessus* (*Mabsc*) ATCC 19977 smooth variant was embedded in agar beads with a diameter of 100–200 µm, and mice were inoculated oropharyngeally with 2 × 10^5^ CFUs per mouse. Histology confirmed that beads of this size lodge in small airway (Supplementary Figure S1). Both control (sterile) and *Mabsc* tryptic soy agar beads (TSA) were prepared using 1.5% of noble agar (BD Difco) in tryptic soy broth (BD Difco). To prepare the beads, *Mabsc* was cultured for 3 days in Middlebrook 7H9 broth base media (Sigma-Aldrich) prepared as described by the manufacturer and supplemented with 10% of Middlebrook ADC (albumin/dextrose/catalase) growth media (Millipore Sigma) and sequentially diluted 1:5 (log phase) in 7H9 media with 10% ADC for ∼24 h. Cultures (1.5 mL) were washed once in 1X PBS with calcium and magnesium, pelleted at 12,000 x g for 5 min, and resuspended in 1.2 mL of saline. To break down the bacterial clumps, washed cultures were sonicated 10 times, one second each, using a CL-188 sonicator (Qsonica). Optical density (OD) was determined using a SPECTRONIC 200E spectrophotometer (Thermo Fisher) with absorbance set to 600 nm. The bacterial suspension was concentrated to an OD of 2.2 with an expected concentration of ∼1 × 10^10^ CFU/mL. The following reagents were equilibrated to 50 °C: 150 mL of heavy mineral oil (Millipore Sigma catalog number: 330760) to a 250 mL Erlenmeyer flask with a stir bar of 6.5 cm, 9 mL of 1.5% TSA agar in a 15 mL tube and 1 mL of *Mabsc* OD 2.2 or 1 mL of saline for the sterile beads. After equilibration time, the flask was placed on a stir plate (Corning PC-620), setting 7, making sure the vortex within the liquid did not reach the stir bar. The 9 mL of 1.5% TSA agar was rapidly added to 1 mL of bacterial suspension or 1 mL of saline and immediately transferred to the stirring oil on the side of the vortex and allowed to stir for 6 min. The flask was placed into ice and stirring continued for 30 additional min at a speed setting to 6. Suspended beads were centrifuged at 3000 x g for 15 min, 12–15 °C. The combined pellets were transferred to a new tube, and beads were washed 3 times with 0.1% triton in saline at 3000 x g for 15 min, 12–15 °C. To obtain the optimal bead size between 100 and 200 µm, resuspended beads in 40 mL of saline were allowed to settle for 2–5 min, and three different layers formed with small beads on the top and large beads at the bottom. The small beads from ∼10 mL from top were discarded, and the middle layer 10–20 mL (100–200 µm) were transferred to a new tube. This procedure was repeated several times to accumulate the correct sized beads and removing those of improper size. To ensure the correct bead size, the beads were observed and measured using the Zeiss Axiovert 40 CFL inverted microscope. After the bead size selection process, two more washes were performed by settling the beads for 15 min, then resuspending in 1X PBS. A final 40%–50% slurry in 1X PBS with calcium and magnesium was obtained. A portion of the slurry was diluted, sonicated, and serial diluted for enumerating CFUs.

### 2.4 Depletion of resident alveolar macrophages with clodronate-loaded liposomes

Mice were administered 50 µL of control or clodronate-loaded liposomes (Liposoma) via oropharyngeal inoculation the day prior to infection with either planktonic or bead-embedded bacteria to deplete resident alveolar macrophages (34) prior to *Mabsc* exposure. Depletion of macrophages and duration of depletion was confirmed by flow cytometric analysis of bronchoalveolar lavage (BAL) cells in pilot studies (data not shown), and following infection.

### 2.5 Lung tissue homogenization to determine bacterial burden

**Figure 1:**
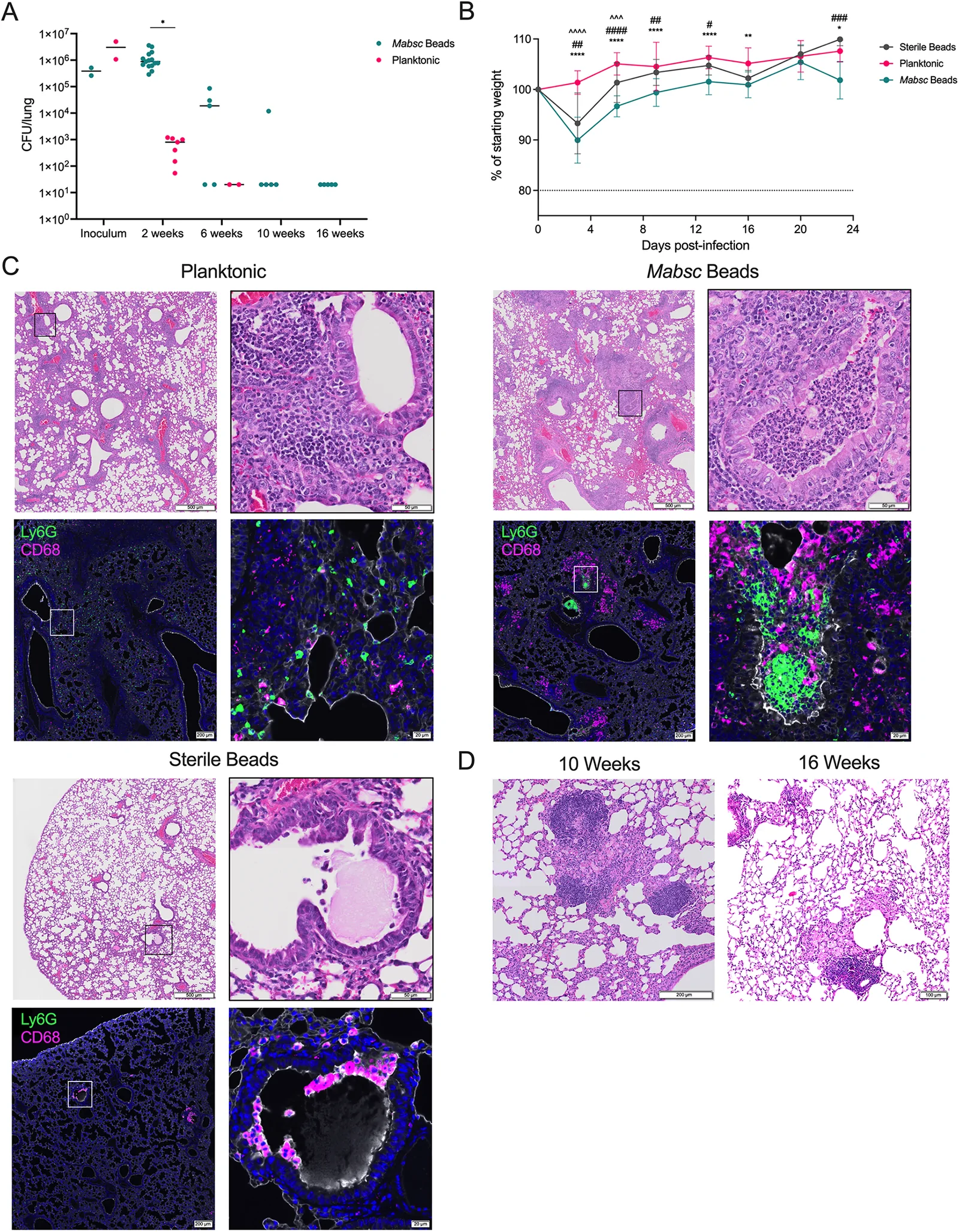
*Mabsc* embedded in agar beads leads to a sustained infection in mice with the development of granulomatous pathology. Mice were inoculated with sterile agar beads, planktonic *Mabsc*, or *Mabsc* embedded in agar beads and harvested at 2, 6, 10, and 16 weeks. **(A)** Right lung tissue was collected from mice harvested at the indicated time points, homogenized, and plated to determine CFUs. **, *p* ≤ 0.01. *N* = 2–15 mice per group per timepoint, 3 pooled independent experiments. *P* values were determined by multiple unpaired t-tests with Holm-Šidák multiple comparisons test. **(B)** Mice were inoculated with sterile agar beads, planktonic *Mabsc*, or *Mabsc* embedded in agar beads and weighed at the indicated time points over the course of 23 days. Significant differences at each time point between sterile beads and planktonic infection are indicated by ^, sterile beads and *Mabsc* beads by #, and *Mabsc* beads and planktonic by *. *, *p* ≤ 0.05; **, *p* ≤ 0.01; ***, *p* ≤ 0.001, ****, *p* ≤ 0.0001. *N* = 3–20 mice per group per timepoint, 2 pooled independent experiments. *P* values were determined by two-way ANOVA with Tukey's multiple comparisons test. **(C)** Lung tissue was collected from mice harvested 2 weeks post-infection, and 5 um tissue slices from paraffin-embedded lung tissue were stained with H&E or using immunofluorescence. Immunofluorescence sections were stained with antibodies against Ly6G (green) to detect neutrophils and CD68 (magenta) to detect macrophages. Scale bars for the H&E sections are 500 µm and 50 µm for the inset, and scale bars for the immunofluorescence are 200 µm and 20 µm for the inset. **(D)** Lung tissue was collected from mice harvested at 10 and 16 weeks post-infection, and 5 um tissue slices from paraffin-embedded lung tissue were stained for H&E. Scale bars are 200 µm (left-hand image in each pair) and 100 µm (right-hand image in each pair).

**Figure 2:**
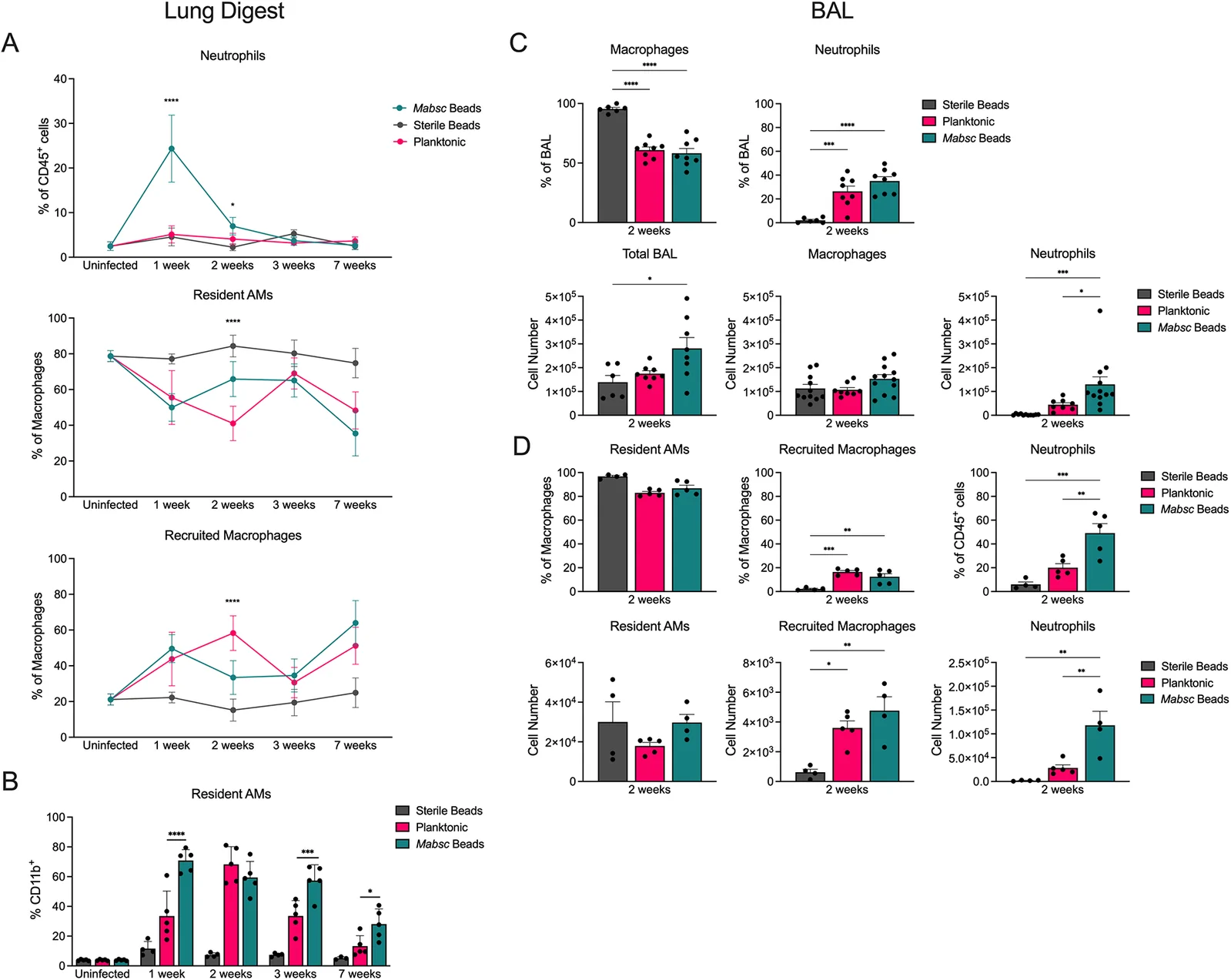
*Mabsc* embedded in agar beads drives early neutrophil recruitment and sustained inflammation. Mice were inoculated with sterile agar beads, planktonic *Mabsc*, or *Mabsc* embedded in agar beads and harvested at 1, 2, 3, and 7 weeks. **(A)** Lung tissue was collected from mice harvested at the indicated time points, homogenized, and stained then analyzed by flow cytometry. Significance asterisks are shown for the comparison between planktonic infection and *Mabsc* bead infection. *, *p* ≤ 0.05; ****, *p* ≤ 0.0001. *N* = 3–10 mice per group per timepoint, 2 pooled independent experiments. *P* values were determined by two-way ANOVA with Tukey's multiple comparisons test. **(B)** Same as in **(A)** Shown is the percent of resident AMs that are CD11b ^+^ . *, *p* ≤ 0.05; ***, *p* ≤ 0.001; ****, *p* ≤ 0.0001. *N* = 4–5 mice per group per timepoint, 2 pooled independent experiments. *P* values were determined by two-way ANOVA with Tukey's multiple comparisons test. **(C)** BAL was collected from mice harvested at 2 weeks, and cytospins were assessed for the number of total BAL cells, macrophages, and neutrophils. Percentages are shown as % of total BAL cells. *, *p* ≤ 0.05; **, *p* ≤ 0.01; ***, *p* ≤ 0.001. *N* = 8–12 mice per group per timepoint, 2 pooled independent experiments. *P* values were determined by one-way ANOVA with Tukey's multiple comparisons test. **(D)** BAL was collected from mice harvested at 2 weeks and stained for flow cytometry to evaluate numbers of resident AMs, recruited macrophages, and neutrophils. Percentages are shown as % of total macrophages or % of CD45^+^ cells (for neutrophils). *, *p* ≤ 0.05; **, *p* ≤ 0.01. *N* = 4–5 mice per group per timepoint, 2 pooled independent experiments. *P* values were determined by one-way ANOVA with Tukey's multiple comparisons test.

**Figure 3:**
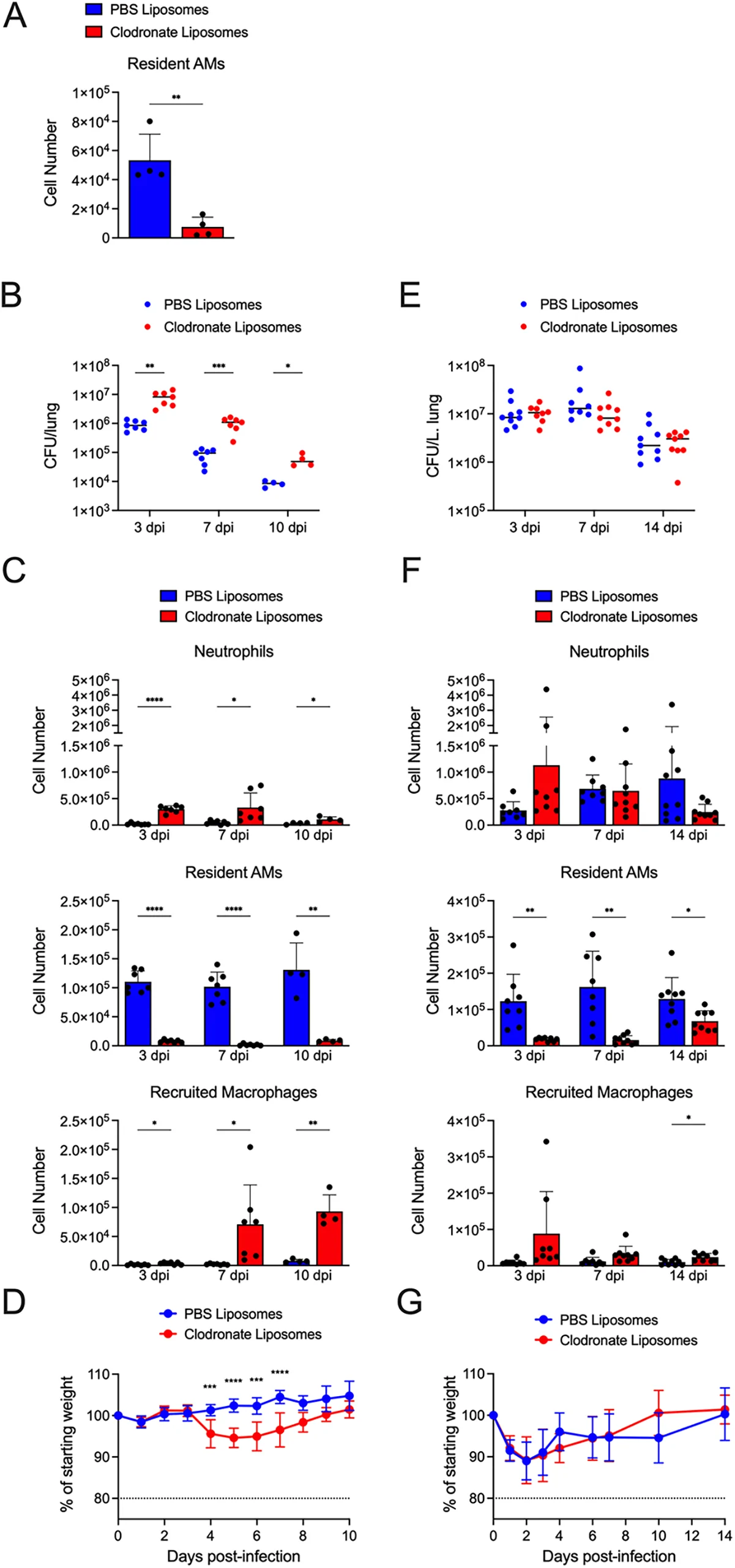
Depletion of RAMs demonstrate that they are key to control of initial alveolar *mabsc* infection but not required for initial control of *mabsc* airways infection. **(A–D)** Mice were treated with control (PBS) or clodronate liposomes 24 h prior to infection with planktonic *Mabsc* and harvested 3, 7, and 10 dpi. **(A)** BAL was collected from mice harvested 24 h after liposome treatment and stained for flow cytometry to assess the number of resident AMs. **, *p* ≤ 0.01. *N* = 4 mice per group, 2 pooled independent experiments. *P* values were determined by unpaired t-test. **(B)** Whole lung tissue was collected from mice harvested at the indicated time points, homogenized, and plated to determine CFUs. *, *p* ≤ 0.05; ***, *p* ≤ 0.001. *N* = 4–7 mice per group per timepoint, 2 pooled independent experiments. *P* values were determined by multiple unpaired t-tests with Holm-Šidák multiple comparisons test. **(C)** BAL was collected from mice harvested at the indicated time points and stained then analyzed by flow cytometry. *, *p* ≤ 0.05; **, *p* ≤ 0.01; ***, *p* ≤ 0.001; ****, *p* ≤ 0.0001. *N* = 4–7 mice per group per timepoint, 2 pooled independent experiments. *P* values were determined by multiple unpaired t-tests with Holm-Šidák multiple comparisons test. **(D)** Mice were weighed daily over the course of the 10-day time course. Dashed line indicates humane endpoint of 80% of starting weight. *, *p* ≤ 0.05; ***, *p* ≤ 0.001; ****, *p* ≤ 0.0001. *N* = 4–18 mice per group per timepoint, 2 pooled independent experiments. *P* values were determined by multiple unpaired t-tests with Holm-Šidák multiple comparisons test. **(E–G)** Mice were treated with control (PBS) or clodronate liposomes 24 h prior to infection with bead embedded *Mabsc* and harvested 3, 7, and 14 dpi. **(E)** Left lung tissue was collected from mice harvested at the indicated time points, homogenized, and plated to determine CFUs. *N* = 8–9 mice per group per timepoint, 2 pooled independent experiments. *P* values were determined by multiple unpaired t-tests with Holm-Šidák multiple comparisons test. **(F)** BAL was collected from mice harvested at the indicated time points and stained then analyzed by flow cytometry. *, *p* ≤ 0.05; **, *p* ≤ 0.01; ***, *p* ≤ 0.001. *N* = 8–9 mice per group per timepoint, 2 pooled independent experiments. *P* values were determined by multiple unpaired t-tests with Holm-Šidák multiple comparisons test. **(G)** Mice were weighed daily over the course of the 14 day time course. Dashed line indicates humane endpoint of 80% of starting weight. *, *p* ≤ 0.05. *N* = 4-26 mice per group per timepoint, 2 pooled independent experiments. *P* values were determined by multiple unpaired t-tests with Holm-Šidák multiple comparisons test.

Lungs (left, right, or whole, as indicated in figure) were homogenized in 1X PBS + 0.1% triton with stainless steel beads (Next Advance) using a bullet blender homogenizer (Next Advance) twice for 3 min at level 7–8. To minimize number of mice used, protocols were devised that processed one lung for CFUs and the other lung for histology and immunofluorescence. Pilot experiments demonstrated that use of one lung for CFU reproducibly quantitated bacterial load, and single lung CFUs were ∼1/2 total lung CFUs. Homogenized lungs were serially diluted and plated for CFUs on LB agar media plates that were incubated in a non-humidified incubator at 37 °C for 4–5 days. Limit of detection for the assay is 20 CFU/lung. Data in Figures 1, 2 were derived from experiments in which planktonic and bead-based *Mabsc* infections were performed in the same experiment, and lungs were processed identically for each type of infection. In Figure 3, planktonic and bead-embedded *Mabsc* infections were performed in different experiments, wherein clodronate liposome and PBS liposome treated mice were inoculated with either planktonic or bead-embedded *Mabsc*.

### 2.6 Flow cytometry

BAL cells were counted via hemocytometer, pelleted, and resuspended in 1X HBSS + 3% FBS containing anti-CD16/32 (Fc Block). For lung digest flow analysis, lung tissue was mechanically dissociated prior to enzymatic digestion using Liberase TM (Roche) for 30 min, and cell homogenates were resuspended in HBSS + 3% FBS containing anti-CD16/32 (Fc Block). Cells were then stained with the following fluorescently conjugated antibodies for 45 min and washed: CD45 (clone 30-F11), Siglec-F (clone E50-2440), F4/80 (clone BM8), CD11b (clone M1/70), CD64 (clone X54-5/7.1), Ly6G (clone 1A8), CD88 (clone 20/70), and CD11c (clone N418). See Supplementary Figure S5 for flow cytometry gating strategy. Samples were acquired on a LSRII, LSRFortessa, or Symphony A5 flow cytometer and analyzed using FlowJo v10 software.

### 2.7 Immunopathology and immunofluorescence staining

In order not to disturb the architecture of the lungs and the location of the beads and associated inflammation, lungs were not instilled with agar or other fixative at the time of harvest. Instead, following euthanasia, the trachea was tied off while lungs were at functional residual capacity, thus sealing air inside the lungs. This maneuver prevented atelectasis/alveolar collapse once the thorax was breached and negative pressure was no longer being exerted on the lungs. If only one lung was used for histologic examination/imaging, the main stem bronchus was tied off before separation from the other lung. Lungs were then submerged in formalin to fix the tissue. Follow fixation, tissues were embedded in paraffin blocks prior to sectioning into 5 µm sections. Sections were then deparaffinized and heat-induced antigen retrieval was performed using Dako Target Retrieval Solution (pH 6.0). Sections were then permeabilized, blocked, and stained with primary antibodies to CD68 (2449C, R&D Systems) and Ly6G (1A8, Biolegend) followed by appropriate secondary antibodies (Invitrogen). Sections were then lectin stained (Vector Laboratories) prior to mounting with VECTASHIELD Vibrance Antifade Mounting Medium with DAPI (Vector Laboratories). Stained slides were imaged using a VS200 microscope (Olympus).

### 2.8 Statistical analysis

All data were analyzed using GraphPad Prism 10 software. Data were evaluated for statistically significant differences using the tests indicated. A *p*-value < 0.05 was considered statistically significant. All differences not specifically indicated to be significant were not significant (*p* > 0.05).

## 3 Results

### 3.1 *Mabsc* embedded agar beads produced prolonged infection that was associated with airway infiltration by neutrophils and macrophages and evolution of granulomatous structures

To compare *Mabsc* clearance and immune responses during alveolar vs. bead-based obstructive small airway infection, mice were inoculated via the oropharyngeal (o.p.) route with either *Mabsc* in suspension (“planktonic *Mabsc*”; 1 × 10^6^ CFU per mouse), *Mabsc* embedded in agar beads (2 × 10^5^ CFU per mouse), or sterile agar beads. The different initial inocula result in roughly equivalent maximal lung bacterial burden, occurring at 3 days post infection. During *Mabsc* planktonic infection, CFUs remain static between inoculation and day 3, and then begin to decline; whereas in *Mabsc* agar bead infection, CFUs increase during the first 3 days after inoculation and then plateau (Supplementary Figure S2). In previous work, we observed that almost all mice infected with this inoculum of planktonic *Mabsc* cleared infection by 3 weeks post-infection, and about half of the mice receiving *Mabsc* beads remained infected at 7 weeks (28). In the current study, lung tissue was harvested for quantification of *Mabsc* bacterial burden and histology at 2 weeks, 6 weeks, 10 weeks, and 16 weeks post-infection. At the 2-week time point, *Mabsc* lung CFUs for the bead-infected mice were similar to CFUs in the inoculum, whereas lung CFUs for the planktonic *Mabsc* infected mice were over 1000-fold lower than the inoculum, demonstrating robust bacterial clearance (Figure 2A). At 6 weeks post-infection, over half of the mice infected with *Mabsc* beads had detectable bacteria at only about 10-fold lower levels than the inoculum (Figure 2A), whereas there were no detectable lung CFUs in mice that had received planktonic *Mabsc*. Most of the bead-infected mice cleared *Mabsc* by 10 weeks while all mice had cleared the bacteria by 16 weeks (Figure 2A). Duration of infection and kinetics of bacterial clearance, with heterogeneity in the rate of *Mabsc* clearance from bead-infected mice, is consistent with data published by others (21). Modified Kinyoun staining of lung tissue isolated two weeks post infection revealed the presence of acid-fast bacilli (AFB) within remnants of infected beads, whereas AFB were absent from planktonic infected mice (Supplementary Figure 1A). At later time points (10 weeks post-infection), AFB remained in lungs of bead-infected mice despite the breakdown of the bead itself (Supplementary Figure 3B).

As a measure of systemic inflammation and stress, we weighed the mice over the course of the first 24 days of infection (Figure 2B). Mice inoculated with planktonic *Mabsc* exhibited no weight loss and continued to gain modest amounts of weight during the first 3 weeks of infection. In comparison, mice inoculated with both sterile beads and *Mabsc* beads experienced 5%–10% weight loss in the first 3 days after bead inoculation, suggesting that airway obstruction alone, independent of infection, stressed the animals. However, mice inoculated with sterile beads quickly recovered and, by day 6, regained sufficient weight to match the weights of mice in the planktonic *Mabsc* cohort. *Mabsc* bead inoculated mice also began to regain weight after day 3 of infection, but their recovery was slower, and they did not achieve equivalent weights to the other 2 cohorts until almost 3 weeks post-infection.

The significant differences in weight loss and bacterial burden observed at 2 weeks post-infection between the planktonic and bead models of *Mabsc* infection were accompanied by two distinct patterns of immune infiltration into the lung (Figure 2C). Hematoxylin and eosin staining of lung sections from mice inoculated with planktonic *Mabsc* revealed diffuse infiltration of inflammatory cells throughout the lung, although the airways were patent. In contrast, lung from the *Mabsc* bead infected mice demonstrated a robust recruitment of inflammatory cells into the airways into which *Mabsc* beads had lodged, and inflammation in the lung parenchyma concentrated around the occluded airways, consistent with our previous findings (26). Immunofluorescent staining indicated that cells surrounding the bead remnants consisted of both Ly6G^+^ neutrophils and CD68^+^ macrophages (Figure 2C). Although both neutrophils and macrophages were present in the lungs of mice that received planktonic *Mabsc*, no areas of focal immune cell concentration were observed. As an additional control to determine the effects of the agar bead on inflammation, we evaluated lung pathology in mice inoculated with sterile agar beads. In contrast to both infections, there was minimal inflammation in the lungs at 2 weeks after sterile bead inoculation, the immune cells recruited to the bead were primarily macrophages, and minimal neutrophils were present in the lung (Figure 2C).

We previously observed granulomatous structures at 7 weeks post-infection (28). Here, we report lung sections from *Mabsc* bead infected mice harvested 10 weeks post-infection also have sustained granulomatous structures, indicating pathology persists after bacterial clearance (Figure 2D). These structures began to resolve by 16 weeks post infection. In contrast, lung sections from planktonic *Mabsc* infected mice harvested 10 and 14 weeks post-infection showed large areas of healthy lung tissue with minor areas of residual inflammation and no obvious granulomatous structures (Supplementary Figure S4).

### 3.2 Relative and absolute abundance of myeloid cell populations varied significantly between *Mabsc* alveolar and bead-based obstructive small airway infections during acute phase of infection

Lung digests were evaluated by flow cytometry to assess relative abundance of immune cell populations in the two infection models during the acute phase of infection (Supplementary Figure S5 and Supplementary Table S1 for flow gating and antibodies used to define different cell populations). Mice inoculated with *Mabsc* beads demonstrated a sharp increase in the relative abundance of neutrophils 1-week post-infection (Figure 3A). Conversely, mice infected with planktonic *Mabsc* exhibited no major increase in neutrophils at any time point but a significant increase in the percentage of macrophages recruited to the lung from the circulation occurred at 2 weeks post-infection, which then subsequently decreased (Figure 3A). Both infection models demonstrated recruitment of macrophages to the lungs during the first week of infection. Between 1- and 2-weeks post infection, there was an increase in the ratio of RAMs (Siglec-F^+^ macrophages) to SiglecF^−^ macrophages (which include pulmonary interstitial and recruited macrophages) in *Mabsc* bead-infected mice. In contrast, the ratio of RAMs to interstitial + recruited macrophages decreased between 1 and 2 weeks in *Mabsc* planktonic infected mice. In lung digests from mice inoculated with sterile beads, frequencies of RAMs, SiglecF- recruited macrophages, and neutrophils remained largely unchanged over the time course evaluated.

Since inoculation with agar beads causes *Mabsc* infection to occur in small airways rather than in alveoli, we questioned whether RAMs, largely found in alveoli (35), were activated during *Mabsc* bead infection. We used flow cytometry to assess cell surface expression of CD11b (36, 37) on RAMs isolated from digested lung tissue over the course of infection (Figure 3B). In mice that received planktonic *Mabsc*, the percentage of RAMs expressing high levels of CD11b increased 1-week post-infection, peaked at 2 weeks, and tapered off (Figure 3B). Despite infection being localized to the airways, in mice that received *Mabsc* beads, the majority of RAMs exhibited high expression of CD11b beginning at 1-week post-infection, which persisted throughout the infection, although the percentage decreased over time (Figure 3B). RAMs in mice that had received sterile beads expressed minimal CD11b expression.

Lung digests reflect total immune cells in the lung, including in the interstitium and submucosa. To better understand which populations of immune cells were recruited into the airspaces, bronchoalveolar lavage (BAL) samples from 2-week post-infection were evaluated (Figure 3C). Cytospins of BAL cells from mice that received planktonic *Mabsc* demonstrated a modest influx of neutrophils, but macrophages remained the predominant population. In contrast, mice infected with *Mabsc* beads had significantly greater absolute neutrophil numbers in the BAL (Figure 3C) at 2-weeks post-infection. BAL cell cytospins from mice administered sterile beads contained almost exclusively macrophages. As cytospin histological evaluation cannot reliably differentiate RAMs and recruited macrophages, we used flow cytometry to categorize BAL macrophages. Both planktonic *Mabsc* and *Mabsc* bead infection caused an increase in recruited macrophages in the BAL at the 2-week time point, which was not seen in mice that received sterile beads (Figure 3D).

### 3.3 RAMs are essential for control of alveolar *Mabsc* infection but not required for initial control of *Mabsc* bead-based obstructive small airways infection

RAMs are a critical first line defense in the lungs against opportunistic pathogens (38, 39). Because of the differences observed in relative abundance of RAMs and activation state of RAMs over the first 3 weeks of infection between planktonic and bead-based *Mabsc* infection, we sought to determine the role of RAMs in control of bacterial burden in the two models of *Mabsc* infection. Oropharyngeal administration of clodronate-loaded liposomes resulted in robust depletion of RAMs within 24 h (Figure 1A). Clodronate liposomes were thus administered 24 h prior to inoculation of mice with either planktonic *Mabsc* or *Mabsc* agar beads. Following infection with planktonic *Mabsc* (1 × 10^6^ CFUs), clodronate-treated mice exhibited 10-fold higher bacterial burdens in the lung at 3 days post-infection as compared to PBS liposome (control) treated mice, which demonstrated no increase in lung CFUs as compared to the inoculum (Figure 1B). Following day 3, clodronate-treated mice could control infection, with lung CFUs decreasing at the same rate as CFUs in the PBS-treated mice; however, the 10-fold higher bacterial burden in the clodronate-treated mice, as compared to PBS-treated mice, was maintained at each time point evaluated (Figure 1B). Flow cytometric analysis of BAL cells demonstrated sustained depletion of RAMs in clodronate-treated mice throughout the duration of the 10-day time course (Figure 1C).

The effect of RAM depletion on the control of *Mabsc* bead-based obstructive small airway infection was then evaluated. Mice were treated o.p. with either PBS liposomes or clodronate liposomes and then inoculated o.*p* 24 h later with *Mabsc* agar beads (2 × 10^5^ CFU/mouse). As before, RAMs remained absent through the first 7 days of infection in clodronate treated mice but began to repopulate the airspaces by day 14 post-infection. No differences in lung bacterial burdens were observed at any time point during the 14-day time course (Figure 1E).

### 3.4 Depletion of RAMs increased infiltration of circulating myeloid cells to the lungs during *Mabsc* alveolar infection, but had minimal effects on immune recruitment to *Mabsc* bead-based obstructive small airway infection

RAMs remained depleted over the 10 days of alveolar infection, yet clodronate-treated mice that received planktonic *Mabsc* infection were able to control infection. To better understand immune cell populations contributing to this control of alveolar infection in the absence of RAMs, BAL cells were analyzed by flow cytometry. Mice treated with clodronate liposomes mounted a robust recruitment of neutrophils by 3 days post infection, which was sustained for 7 days before decreasing by 10-day post infection (Figure 1C). Circulating monocyte-derived macrophages were also recruited to the airspaces in clodronate-treated mice following infection but with more delayed kinetics than seen with neutrophils. There were minimal recruited macrophages present in the BAL at day 3, numbers increased substantially by 7 days post-infection, and were maintained at day 10 (Figure 1C). Control PBS liposome-treated mice, which maintained their RAMs, recruited minimal neutrophils and macrophages to the airways over the course of the 10-day alveolar *Masbc* infection. Mirroring the kinetics of neutrophil infiltration into the lungs, clodronate-treated mice exhibited a modest but significant weight loss beginning at 3 days post-infection, and weight began to recover by day 7 (Figure 1B). PBS liposome-treated mice, as seen with mice not administered any liposomes (Figure 3A), did not lose weight following o.p. inoculation with planktonic *Mabsc*, consistent with the lack of inflammatory cell recruitment to the lungs (40, 41).

Myeloid cell recruitment to the lungs following RAM depletion by o.p. clodronate liposomes was also assessed during *Mabsc* bead-based obstructive small airway infection. As seen with mice that had not received any liposomes (Figure 3), PBS liposome-treated mice inoculated with *Mabsc* agar beads experienced marked recruitment of neutrophils to the lung, with approximately 1 log more neutrophils in the BAL from mice receiving *Mabsc* agar bead infection compared with mice receiving planktonic *Mabsc* infection (Figures 1F, C). Pre-treatment of mice with clodronate liposomes to deplete RAMs prior to *Mabsc* agar bead infection did not alter the magnitude of the neutrophil influx (Figure 1F). Similarly, there was no differences in weight between control and clodronate-treated mice during *Mabsc* agar bead infection (Figure 1G). Despite no difference in bacterial CFUs noted over 14 days in PBS-treated and clodronate-treated mice inoculated with *Mabsc* agar beads, there was a modest but significant increase in macrophages recruited to the lung airspaces in the clodronate-treated mice, as compared to control mice (Figure 1F).

## 4 Discussion

*Mabsc* pulmonary infections in people with chronic airways diseases are increasing in prevalence (6, 42–44), but current antimicrobial therapies are lengthy, challenging to endure, and are often ineffective at eradicating infection (2, 7, 45). A better understanding of why chronic airways disease increases susceptibility to *Mabsc* pulmonary infections will help inform new host-directed therapies. We hypothesized that deposition of inhaled *Mabsc* in obstructed airways, as occurs during chronic airways disease including bronchiectasis, rather than in the alveoli, changes the nature of the initial immune response, and thus might help explain why people with bronchiectasis and chronic lung disease are at increased risk of *Mabsc* chronic infection. We thus compared early host responses to alveolar infection produced by o.p. inoculation with planktonic *Mabsc*, with early responses to *Mabsc* obstructive small airway infection produced by o.p. instillation of *Mabsc* embedded in agar beads. Our studies demonstrated that alveolar *Mabsc* infection elicits a qualitatively different host response than agar bead *Mabsc* infection, with a fundamental difference between the two immune responses being the role of RAMs in control of infection. Not surprisingly, RAMs were of key importance for initial control of bacterial replication during alveolar infection; however, despite RAM activation during bead-based obstructive small airway infection (as detected by upregulation of CD11b), RAMs were not required for initial control of *Mabsc* infection in the airways during the first 14 days of infection. Additionally, despite *Mabsc* agar beads stimulating a robust recruitment of neutrophils and monocyte-derived macrophages to the airways, which greatly surpassed myeloid cell recruitment observed during alveolar *Mabsc* infection, airway *Mabsc* persisted for weeks after alveolar infection had been eradicated.

The marked differences in bacterial CFUs at 2 weeks post-infection could account for some of the differences in the myeloid cell responses observed in planktonic vs. agar bead *Mabsc* infection, especially the increased number of neutrophils recruited to the lungs in the agar bead model. However, the absolute CFUs in the airways at both 1- and 2- weeks post-*Masbc* agar bead infection were similar to the inoculum delivered to mice that received planktonic *Mabsc* infection. Thus, absolute CFUs alone did not drive the neutrophil response. Persistence of high bacterial burden, which occurs in the agar bead model (Figure 2A) (21, 28), may have stimulated the more robust neutrophilic recruitment seen during bead-based obstructive small airway infection. Notably, despite the increased numbers of neutrophils, higher CFUs of bacteria persisted in the bead model.

Two unexpected observations about lung macrophages suggest that lung compartment, and not magnitude of bacterial CFUs, may be a key driver of differential immune responses generated by alveolar vs. bead-based obstructive small airway infections. First, RAMs in the *Mabsc* bead infection exhibited an intense increase in CD11b expression, indicating inflammatory activation (36, 37), despite infection occurring in the airway and not the alveoli and despite RAMs depletion not altering bacterial burden during the first two weeks of infection in *Mabsc* agar bead model. RAM activation could result from debris and bacteria being shed from degraded beads filtering down into the alveoli. Alternately, RAM activation may reflect the overall inflamed state of the lungs, with RAMs being activated by cytokines from epithelial cells or recruited neutrophils.

Another unexpected finding was the difference in relative abundance of total lung digest macrophage populations (resident vs. interstitial + recruited) in planktonic vs. agar bead *Mabsc* infection at 2 weeks post-infection, with a higher resident to recruited ratio in *Mabsc* bead infection (60:40) and an inverted ratio (40:60) in *Mabsc* planktonic infection. A lower relative abundance of RAMs in total lung digest at week 2 of planktonic infection could have resulted from increased recruitment of monocyte-derived macrophages to the lung and/or loss of RAMs due to cell death. This difference in relative abundance of macrophage populations appeared to have been driven by changes in the lung parenchyma and not in lung lumen/airspaces. The majority of macrophages in the lung lumen (as measured in the BAL) in both infection models were RAMs, with recruited macrophages representing only about 10%–20% of total macrophages, and the ratio of resident to recruited macrophages in the BAL was not significantly different between the two models.

The findings presented here add to a body of literature supporting a key role of macrophages in controlling *Mabsc* infections. Clodronate liposomes have been used in several non-mammalian models of *Mabsc* infection to deplete all phagocytes, demonstrating the importance of macrophages in control of *Mabsc* infection. These models have included *Mabsc* intrathoracic infection of *Drosophila melanogaster* (46)*,* as well as both *Mabsc* mono-infection of zebrafish (47, 48) and secondary *Mabsc* infection of zebrafish in which primary *M. marinum* infection had generated granulomas (49). In each of these models, macrophage depletion resulted in uncontrolled replication of *Mabsc,* often associated with death of the organism, highlighting the critical nature of macrophages for control of *Mabsc* infection.

In mice and other mammals, however, there are distinct and unique populations of macrophages for which phenotypes are highly influenced by the local tissue environments (38, 50). Thus, the specific macrophage population(s) that are crucial for control of *Mabsc* in mammals likely depends on the location of infection. At least two prior studies have evaluated the importance of RAMs in control of murine alveolar *Mabsc* infection. One study employed intratracheal clodronate liposomes to deplete RAMs prior to *Mabsc* alveolar infection. This study used intranasal inoculation of *Mabsc* (which results in alveolar deposition of bacteria) rather than o.p. or intratracheal infection, and ATCC 19977 rough morphotype *Mabsc* rather than the smooth *Mabsc* morphotype employed in our study and other studies noted. At day 5 post infection, the authors found that depletion of RAMs resulted in increased *Mabsc* CFU in the lung during alveolar infection (51). Clodronate-treated mice infected with rough *Mabsc* morphotype exhibited a more modest increase in lung CFUs, as compared to control-treated mice, than what we observed with smooth *Mabsc* morphotype (about half a log). Another group used a genetic approach (a combination of diphtheria toxin receptor, Cre-loxP, and Flp-FRT recombination) to selectively deplete RAMs prior to *Mabsc* alveolar infection (52), and these investigators also concluded that RAMs are required for initial control of *Mabsc* infection (52). In that study, mice lacking RAMs exhibited no difference in lung CFUs compared to control mice at day 3 of infection, a modest increase in CFUs at day 7 (<1 log), and no difference in CFUs at day 10. Mice received a higher inoculum than in our study (5 × 10^7^ CFU/mouse in their study vs. 1 × 10^6^ CFU/mouse in our study), and the preparation of the bacteria for inoculation also differed. These results suggest that the amount of bacteria in the inoculum, the *Mabsc* morphotype (rough vs. smooth), and the growth state of *Mabsc* influence how RAMs interact with *Mabsc* in the alveoli during the first two weeks of infection.

Although it has been shown that some RAMs remain sessile in the alveoli (53), while others patrol the airspaces (39), the question of whether RAMs traffic into small airways remains poorly understood. In addition, studies have shown that RAMs can influence phagocytic activity and inflammatory state of bronchial epithelial cells (54, 55), and thus may influence host responses in the airways whether or not they traffic there. Herein, we evaluated whether depletion of RAMs influenced infection occurring in the airways, rather than in the alveoli. We found that depletion of RAMs did not alter bacterial burden during the first 14 days of *Mabsc* bead-based obstructive small airway infection, in contrast to the key role of RAMs in controlling *Mabsc* infection in the alveoli. As our study did not characterize beyond 2 weeks of infection, it is possible that depletion of RAMs could influence evolution of infection dynamics at later timepoints, either altering bacterial burden or extent of inflammation. Other than the depletion of RAMs, the only difference observed between PBS liposome-treated mice infected with *Mabsc* agar beads and clodronate liposome-treated mice infected with *Mabsc* agar beads was a modest increase in monocyte derived macrophages recruited to the airspaces (in the BAL) in clodronate-treated mice. As BAL samples the entire lung lumen, including airways and alveoli, this may reflect monocyte-derived macrophages trafficking into the alveoli in response to clodronate-induced apoptosis of RAMs (56) and may not indicate that RAMs influence recruitment of monocyte-derived macrophages to *Mabsc* obstructive small airway infections resulting from inoculation with agar beads.

There are several limitations to our studies to be noted. First, studies are limited by a focus on the early time points in both infection models as well as a focus on myeloid cells. The marked difference in control of bacterial CFUs in the two models during the first 2 weeks of infection indicated that key early host defence mechanisms are absent or have failed in the *Mabsc* agar bead model that are functional in the *Mabsc* planktonic model. As demonstrated in data from PBS liposome-treated mice in Figure 1 and in our previous publication (28), immunocompetent mice with intact RAMs control bacterial replication within the first 3 days of infection and begin clearance of bacteria within the first week. In contrast, there is growth of *Mabsc* during the first 3–7 days of agar bead infection, and subsequent bacterial clearance is much delayed compared to rates of elimination of bacteria during planktonic infection (Supplementary Figure S2). We thus focused these studies on the first 10–14 days of infection. Lymphocytes were essentially absent from the BAL during the first week of infection and became a more prominent population in the BAL and lung homogenates in both models as infection progressed (data not shown), and they participated in granuloma formation at late time points in the *Mabsc* agar bead model (Figure 2). Because we were focusing on early events, we did not characterize differences in lymphocyte populations for this study. Second, in the experiments in which clodronate was administered to deplete RAMs, we did not verify histologically the location of residual RAMs, and thus did not rule out the possibility that residual RAMs (< 10% of initial RAMs) may have trafficked to bead-adjacent occluded airway lesions. Finally, the agar bead model relocates infection from alveoli to airways by agar beads lodging in and occluding small airways. Our studies do not differentiate whether the immune response described in the agar bead model is due solely to infection being present in the airway (vs. the alveoli) or due to impaired mucociliary clearance, epithelial cell stress, and/or local hypoxia that may be caused by airway obstruction. However, individuals with chronic airways disease and bronchiectasis often experience obstructed small airways with associated epithelial cell damage, airway inflammation, and impaired mucociliary clearance (57). Data presented here indicate that the immune cells infiltrating to the airspaces during *Mabsc* agar bead infection are similar to sputum cells from people with bronchiectasis, composed primarily of neutrophils and recruited monocyte-derived macrophages (9, 11, 12). Future studies will further characterize the airway milieu in the *Mabsc* agar bead model to determine if it does in fact reflect multiple features of bronchiectasis, with a focus on changes in epithelial cell function, as well as changes in pH and oxygen tension in the airway lumen.

Despite these limitations, our studies highlight two important concepts. First: the lung is an organ with multiple compartments, including alveoli and airways, as well as interstitium and vasculature. Infection can occur uniquely in each different compartment. Alveoli and airways are contiguous sections of the lumen of the lung; however, the immune responses to infections in these two compartments are overlapping but distinct. In human airways diseases with chronic infections such as bronchiectasis, studies have found that immune cells located in the airway lumen are largely recruited from the blood, and RAMs are minimally represented or absent in lung samples from patients (9, 12, 58), findings that mirror our observations here for murine airway *Mabsc* infection. These results highlight the need to consider whether a specific animal model (species, location of infection) used to study *Mabsc* infections adequately reflect immune responses that are relevant in human *Mabsc* lung disease.

A second key concept is that macrophages in the mammalian host are heterogeneous. Macrophage phenotypes are shaped by local tissue environments, pathogen associated molecular patterns (PAMPs), damage associated molecular patterns (DAMPs), the presence of dead host cells, and many other stimuli (38, 50, 59–61). The two murine models of *Mabsc* characterized here demonstrated significant differences in the composition of macrophage populations recruited to the infection and the roles of different macrophage subtypes required for control of bacterial replication. Understanding how the host environment alters bacterial transcriptional patterns, metabolism, and growth is essential for devising novel anti-microbial therapies. The failure of current antimicrobial regimens to eradicate many chronic infections, including *Mabsc*, is thought to be due, in large part, to *in vitro* antimicrobial sensitivity assays not reflecting how bacteria exist *in vivo* (45, 62–64). Knowledge of the macrophage subtype(s), and the host environment generally, present at the site of *Mabsc* infection *in vivo* in the human lung is thus essential when characterizing host-pathogen interactions *in vitro*. Further characterization of the *Mabsc* agar bead model will reveal additional information about the immune niche in damaged and inflamed airways that will hopefully inform development of novel host-directed therapies to help eradicate recalcitrant chronic *Mabsc* lung disease.

## Data Availability

The raw data supporting the conclusions of this article will be made available by the authors, without undue reservation.
