# Supplementary material for: Differential roles of resident lung macrophages during control of murine alveolar and airway Mycobacterium abscessus infection
Source: Front Tuberc. 2026 Jun 11;4:1781507. doi: 10.3389/ftubr.2026.1781507 (PMC13491439; doi:10.3389/ftubr.2026.1781507)
Supplement: Supplementary file 1 [file Supplementaryfile1.docx]

Supplementary Material

**Supplemental Table 1. Antibody targets for flow cytometry analysis.**

| Antigen | Cell Population Defined within Flow Panel |
| --- | --- |
| CD45 | Hematopoietic cells |
| Ly6G | Neutrophils |
| F4/80 | Pan-macrophages |
| CD88 | Pan-macrophages |
| CD64 | Pan-macrophages |
| Siglec-F | RAMs |
| CD11c | RAMs |
| CD11b | Recruited macrophages, activated RAMs, interstitial macrophages |

**Supplemental Figure 1. Planktonic *M. abscessus* deposits in alveoli and *M. abscessus* in agar beads lodges in small airways.** Mice were inoculated with planktonic *Mabsc* or *Mabsc* embedded in agar beads and BAL and lungs were harvested. (A) At day 1 post infection with planktonic *Mabsc*, right lungs were lavaged to collect bronchoalveolar lavage fluid, and left lungs were homogenized. BAL and lung homogenates were plated for bacterial CFUs. (B) At day 3 post infection with planktonic *Mabsc*, cytospins of BAL were performed and stained with modified Kinyoun stain for acid fast bacilli (AFB). Images revealed essentially almost all cells were resident alveolar macrophages, and many contained AFB. (C) At day 6 post infection with *Mabsc* agar beads, lungs were harvested at functional residual capacity, fixed, and paraffin-embedded for histology. Sections were stained with modified Kinyoun stain. The image shows two small airways in which agar beads were lodged (bead in top middle/right airway was lost during processing; bead in bottom right airway contains AFB and is surrounded by leukocytes), and two airways that remain patent (on far left of image, top and bottom).

**Supplemental Figure 2. Time course of planktonic vs. bead CFUs.** Comparison of lung CFUs for planktonic and agar bead *Mabsc* infection over 16 weeks of infection. This data is a compilation of the data in Figures 1 and 3 as well as the data found in PMID: [39589263](https://pubmed.ncbi.nlm.nih.gov/39589263/).

**Supplementary Figure 3.** **Imaging of bacteria in lung sections and lung pathology at late time points during *Mabsc* bead infection.** Mice were inoculated with planktonic *Mabsc* or *Mabsc* embedded in agar beads and harvested at 2, 6, 10, and 16 weeks. Lung tissue was collected from mice harvested 2 weeks post-infection, and 5 um tissue slices from paraffin-embedded lung tissue were modified Kinyoun stained for acid fast bacilli (AFB). (A) Comparison of lung sections from mice 2 weeks post-infection with planktonic (top) or bead embedded (middle, bottom) *Mabsc*. Scale bars are 500 µm and 100 µm for the inset (top), 200 µm and 50 µm for the inset (middle), and 100 µm and 50 µm for the inset (bottom). (B) Lung sections from *Mabsc* bead infected mice 6 weeks (top, left), 10 weeks (top, right) and 16 weeks (bottom) post-infection. Scale bars are 10 µm (top, left), 20 µm (top, right), and 20 µm (bottom).

**Supplementary Figure 4. Inflammation persists in planktonic *Mabsc* infected mice following clearance of bacteria.** Mice were inoculated with planktonic *Mabsc* and harvested at 10 and 14 weeks. Lungs were collected from mice, and 5 um tissue slices from paraffin-embedded lung tissue were H&E stained. Shown are representative images from 10 weeks (left) and 14 weeks (right). Scale bars are 100 µm for both panels.

**Supplemental Figure 5. Flow cytometry gating strategies.** (A) Shown is the flow cytometry gating strategy used to analyze lung digest samples. Recruited AM gate is CD11b^+^ and may also include IMs. (B) Same as in (A) but for BAL samples. Recruited AM gate is CD11b^+^ but should not include IMs.
